# Study of the Antioxidant Properties of *Filipendula ulmaria* and *Alnus glutinosa*

**DOI:** 10.3390/plants11182415

**Published:** 2022-09-16

**Authors:** Stanislav Sukhikh, Svetlana Ivanova, Liubov Skrypnik, Alina Bakhtiyarova, Viktoria Larina, Olesia Krol, Alexander Prosekov, Andrej Frolov, Maria Povydysh, Olga Babich

**Affiliations:** 1 Institute of Living Systems, Immanuel Kant Baltic Federal University, A. Nevskogo Street 14, 236016 Kaliningrad, Russia; stas-asp@mail.ru (S.S.); lskrypnik@kantiana.ru (L.S.); bakhtiarova.allina@yandex.ru (A.B.); surinac@mail.ru (V.L.); ole-jolie@yandex.ru (O.K.); olich.43@mail.ru (O.B.); 2 Natural Nutraceutical Biotesting Laboratory, Kemerovo State University, Krasnaya Street 6, 650043 Kemerovo, Russia; 3 Department of General Mathematics and Informatics, Kemerovo State University, Krasnaya Street, 6, 650043 Kemerovo, Russia; 4 Laboratory of Biocatalysis, Kemerovo State University, Krasnaya Street 6, 650043 Kemerovo, Russia; a.prosekov@inbox.ru; 5 K.A. Timiryazev Institute of Plant Physiology RAS, Botanicheskaya Uliza 35, 127276 Moscow, Russia; frolov@ifr.moscow; 6 Department of Biochemistry, Saint Petersburg State Chemical Pharmaceutical University, Professora Popova 14A, 197376 Saint Petersburg, Russia; maria.povydysh@pharminnotech.com; 7 Department of Pharmacognosy, Saint Petersburg State Chemical Pharmaceutical University, Professora Popova 14A, 197376 Saint Petersburg, Russia

**Keywords:** aging, *Filipendula ulmaria*, *Alnus glutinosa*, antioxidant activity, free radicals, reactive oxygen species

## Abstract

The demographic situation of the last few decades is characterized by the increased numbers of elderly and senile people, i.e., by the aging of the population. In humans, ageing is closely associated with the enhanced production of reactive oxygen species (ROS), development of systemic inflammation and related vascular atherosclerotic alterations and metabolic disorders, like obesity, diabetes mellitus and neurodegenerative diseases. As these age-related alterations are directly associated with up-regulation of ROS production and development of chronic oxidative stress, their onset can be essentially delayed by continuous daily consumption of dietary antioxidants—natural products of plant origin. Such antioxidants (in the form of plant extracts, biologically active complexes or individual compounds) can be supplemented to functional foods, i.e., dietary supplementations for daily diet aiming prolongation of active life and delay of the senescence onset. Thereby, use of widely spread medicinal plants might essentially improve cost efficiency of this strategy and availability of antioxidant-rich functional foods. Therefore, here we addressed, to the best of our knowledge for the first time, the antioxidant activity of the extracts prepared from the aerial parts of *Filipendula ulmaria* and *Alnus glutinosa* growing in the Kaliningrad region of Russia, and assessed the contents of the biologically active substances underlying these properties. It was found that the extract prepared with the leaves of *Filipendula ulmaria* and female catkins of *Alnus glutinosa* demonstrated high antioxidant activity, although the former plant was featured with a higher antioxidant potential. The highest antioxidant activity detected in the methanol extracts of *Alnus glutinosa* reached 1094.02 ± 14.53 µmol TE/g, radical scavenging of activity was 584.45 ± 35.3 µmol TE/g, reducing capacity at interaction with iron complex—471.63 ± 7.06 µmol TE/g. For the methanol extracts of Filipendula ulmaria the antioxidant activity reached 759.78 ± 19.08 µmol TE/g, antioxidant activity for free radical removal was 451.08 ± 24.45 µmol TE/g and antioxidant activity for restorative ability with iron complex was 332.28 ± 10.93 µmol TE/g. These values are consistent with the total yields of the extracts and their content of ellagic acid. The ethyl acetate extracts of the both plants showed just minimal antioxidant activity. Thus, the considered extracts have an essential potential. This creates good prospects for the further use of herbal extracts of *Filipendula ulmaria* and *Alnus glutinosa* as a source of natural antioxidants.

## 1. Introduction

The demographic situation of recent decades is characterized by an increase in the numbers of elderly and senile people, i.e., by aging of the world population. According to the forecasts of the United Nations (UN), the age group of people over 60 years old will keep growing and will exceed 1 billion people by 2025. Increasing human life expectancy, reducing morbidity, and preventing premature aging are the most important tasks of modern preventive medicine [1]. Population aging is associated with an increase in the number of diseases, primarily such as atherosclerosis, coronary heart disease, arterial hypertension, chronic heart failure, diabetes mellitus [2]. The cardiovascular system is the first to be involved in age-related changes, when the others are still not affected. It is the cardiovascular system that primarily determines the perspective: longevity or early senility. As these age-related alterations are directly associated with up-regulation of reactive oxygen species (ROS) production and development of chronic oxidative stress, their onset can be essentially delayed by continuous daily consumption of dietary antioxidants—natural products of plant origin. Currently, this strategy is recognized as one of the most important elements in counteracting premature aging [3,4]. In this regard, finding new sources of natural antioxidants that prevent the age-related alterations in the human body is an urgent task [5]. Such antioxidants (in the form of plant extracts, biologically active complexes or individual compounds) can be supplemented to functional foods, i.e., dietary supplementations of daily diet aiming prolongation of active life and delay of the senescence onset. Thereby, the use of widely spread (and, therefore, readily available) medicinal plants might essentially improve cost efficiency of this strategy and availability of antioxidant-rich functional foods.

Dietary and therapeutic antioxidants are mostly isolated from crop and medicinal plants [6,7]. In addition, the industry involved in the processing of agricultural by-products is also a potentially important source of natural antioxidants [8]. These natural antioxidants from plant materials are primarily polyphenols (phenolic acids, flavonoids, anthocyanins, lignans, stilbenes), carotenoids (lutein and carotene), and vitamins (vitamins E and C) [9]. In general, the natural antioxidants, especially polyphenols and carotenoids, have a wide range of biological effects, such as anti-inflammatory, antibacterial, antiviral, anti-aging, and anticancer [10,11,12,13,14,15]. The major health benefits of antioxidants attract considerable attention in the field of nutrition to practical methods for isolation of natural antioxidants, adequate assessment of antioxidant activity, and their key sources in crop and medicinal plants [16]. Numerous medicinal plants possess antioxidant activity [17]. Preparations from *Filipendula ulmaria* and *Alnus glutinosa*, which are widely spread in the Eastern Baltic and have been known for their therapeutic properties since ancient times [18,19], are of particular interest in this regard. The need for exploratory studies to identify vicariant *Filipendula ulmaria* and *Alnus glutinosa* that are similar in chemical composition is brought on by the demand for the use of officinal species as promising medicines [20] and the concurrent insufficiency of such raw materials in Russia. The phytochemical study of the species *F. ulmaria* and *A. glutinosa* growing in the Kaliningrad region is an important agenda in this regard.

*Filipéndula Mill* (meadowsweet) is a genus of about 30 perennial herbaceous plants in the Rosaceae family, some of which grow in Russia (Western and Eastern Siberia, the European part of Russia, the Southern Urals, Bashkiria, the Far East, the upper Dnieper region, the Volga-Kama, Verkhne-Volzhsky districts). Among the most common is *Filipendula ulmaria* (L.) Maxim, an official plant species in many countries. According to studies, salicylic acid derivatives [17], 2-pyrone-4,6-dicarboxylic acid [21], phenylpropanoids [22], flavonoids [23], tannins [24], and essential oils [25] were found in *F. ulmaria* growing in Western Siberia. Phenolic components are active compounds that determine the presence of immunostimulatory [26], antimicrobial [27], antiallergic [28], and other types of activity in *F. ulmaria* preparations.

The antioxidant properties of *F. ulmaria* and *A. glutinosa* growing in the Kaliningrad region (the western outskirts of the Russian Plain, bordered in the north and east by Lithuania, in the southwest by Poland, from the west the territory of the region is washed by the waters of the Baltic Sea and its freshwater bays—Curonian and Kaliningradsky) were investigated [29,30]. The influence of air masses formed in the Atlantics and the Eurasia continent ensures the temperate continental climate in the Kaliningrad region. The position of the region on the southeastern coast of the Baltic Sea is also important. It is known that the number of species of the local wild-growing flora reaches 1436, and the number of species of the ornamental tree and shrub flora—699 taxa, which indicates a high degree of saturation with species in a relatively small area (15000 km^2^). However, the quantitative and qualitative characteristics of the species *F. ulmaria* and *A. glutinosa*, as well as their antioxidant activity, have not been sufficiently studied [29,30].

For this reason, a hypothesis was proposed that the extracts prepared from the *F. ulmaria* and *A. glutinosa* plants, growing in the Kaliningrad region, might have high antioxidant activity. If this hypothesis can be confirmed, the Kaliningrad region could be considered as a promising territory for culturing these plants with pharmaceutical purposes. Therefore, her we address, to the best of our knowledge for the first time, the antioxidant potential of these plants. For this, we studied the antioxidant properties of aqueous, aq. ethanolic, methanolic and ethyl acetate extracts prepared from the aerial parts of the *F. ulmaria* and *A. glutinosa*, plants growing in the Kaliningrad region of Russia.

## 2. Results

### 2.1. Total Yield of Extracts

At the first step the total extraction yields were assessed (Table 1). The data indicated that the extraction with ethyl acetate delivered relatively low yields. Thus, relatively low amounts of hydrophobic metabolites could be recovered from the plant material, although the optimal extraction conditions (temperature, extraction period, and extractant volumes) were selected based on the literature search [31]. The maximal total extract yield for *A. glutinosa* (cones) was observed in the methanol extraction. In the case of *F. ulmaria* (leaves), extraction with 70% (*v*/*v*) aq. ethanol turned to be the most efficient.

### 2.2. Contents of Phenolic Compounds

**Figure 1:**
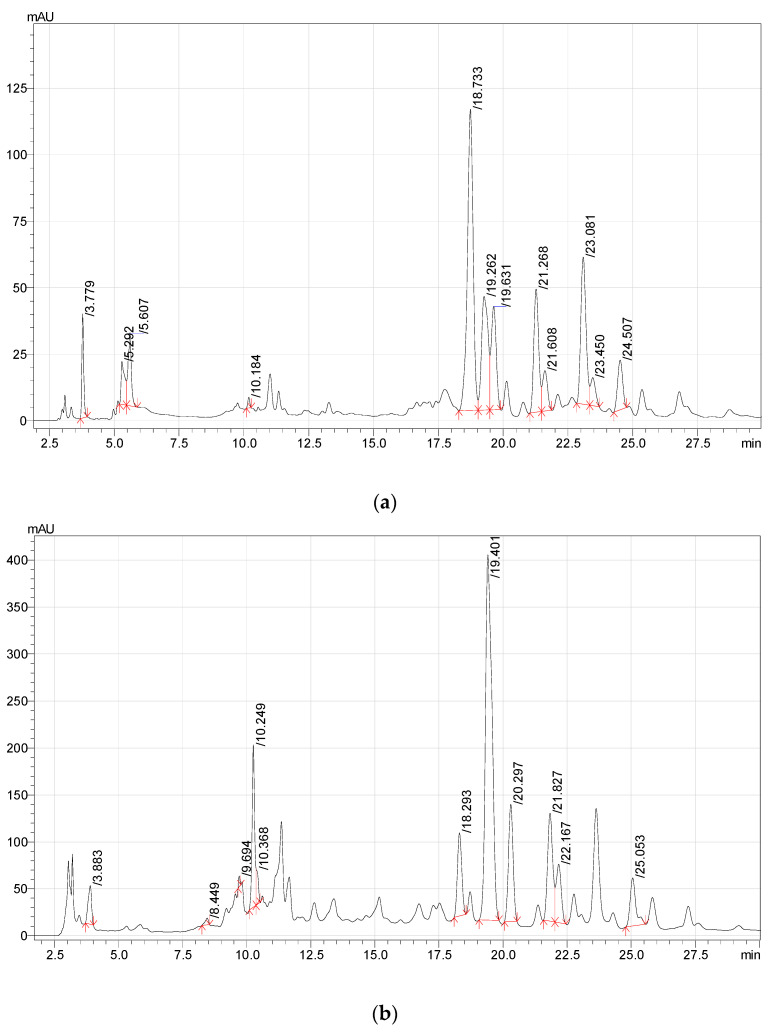

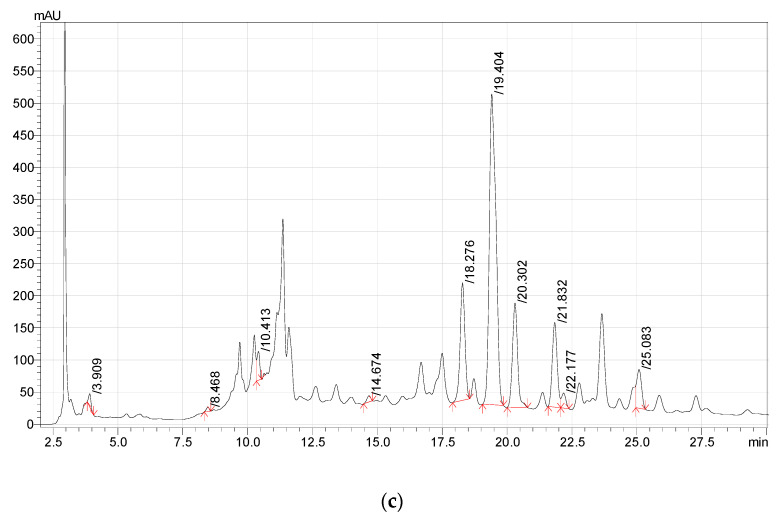
RP-HPLC-UV chromatograms acquired at 254 nm of ethyl acetate (**a**), methanol (**b**), and aqueous ethanolic extracts (**c**) of *F. ulmaria* (leaves).

RP-HPLC analysis revealed high contents of phenolic compounds in the *F. ulmaria* extracts (Table 2, Figure 1). Thereby, 3,4-dihydroxybenzoic acids and rutin were found only in ethyl acetate extract (Figure 1A), catechin—only in the methanol extract (Figure 1B). Quercetin-3D-glucoside, luteolin-7-glucoside, hyperoside, and astragalin were found in all three *F. ulmaria* extracts, but the aq. ethanolic extract was the richest in these compounds (Figure 1). Gallic acid was also presented in all *F. ulmaria* extracts, although its highest contents were found in the methanolic extract.

**Figure 2:**
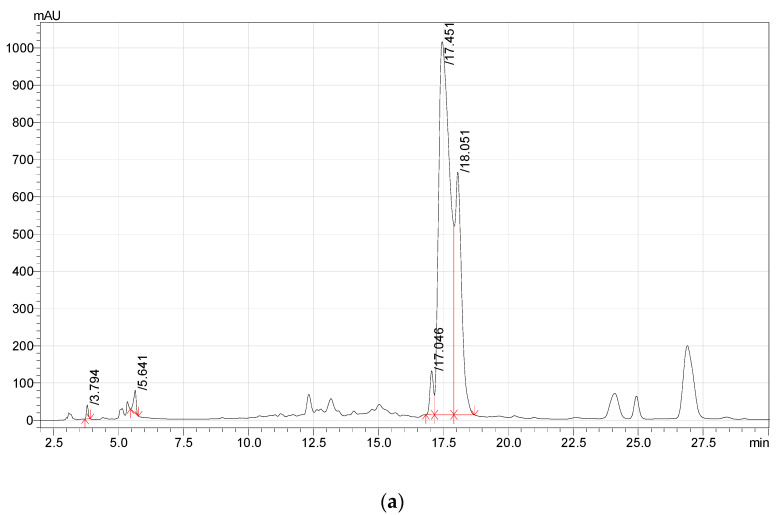

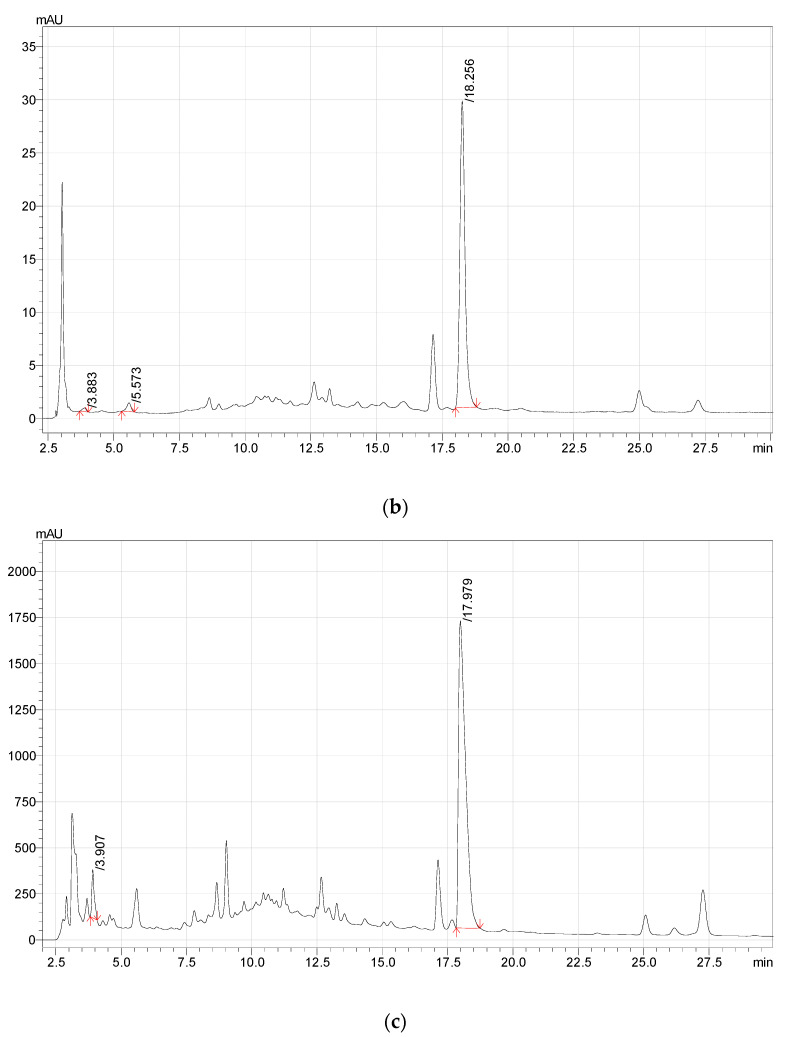
HPLC chromatogram of (**a**) ethyl acetate, (**b**) methanol (1/100 dilution), and (**c**) water extracts of *A. glutinosa* (cones).

*A. glutinosa* extracts did not contain as broad a range of phenolic compounds (Table 1, Figure 2) as the *F. ulmaria* isolates. Ellagic acid was the most abundant component of *A. glutinosa* cone extracts (Figure 2). This metabolite accounted from 10.994 to 44.883 g/kg (Table 1). Moreover, gallic acid was identified in all extracts, and both ethyl acetate and methanol extracts contained trace amounts of 3,4-dihydroxybenzoic acid.

Table 2 demonstrates the content of phenolic compounds in *F. ulmaria* and *A. glutinosa* extracts.

### 2.3. Antioxidant Activity of the A. glutinosa and F. ulmaria Extracts

For analysis of the antioxidant activity, slightly different extraction strategy was applied for the two analyzed plants. The leaves of *F. ulmaria* were extracted with 70% (*v*/*v*) aq. ethanolic solution, ethyl acetate, and methanol, while for the *A. glutinosa* cones, H_2_O, ethyl acetate, and methanol were chosen. In our decision, we relied on the work of Rajbhar and co-authors [32], who showed that hydrophilic polyphenols, including flavonoid aglycones and flavonoid glycosides, which are known to exhibit antioxidant properties, were extracted with water, polar organic solvents (such as methanol, ethanol, acetonitrile and acetone), or their aqueous mixtures. It is known from the literature that, depending on their solubility, these compounds can be efficiently extracted from liquid isolates with ethyl acetate or aqueous mixtures. In preliminary tests, it was found that antioxidant polyphenols of *F. ulmaria* exhibiting antioxidant properties were most efficiently extracted with 70% (*v*/*v*) aq. ethanol, ethyl acetate and methanol, while polyphenols of *A. glutinosa* were extracted with H_2_O, ethyl acetate and methanol.

The extracts of *F. ulmaria* obtained with different organic solvents demonstrated essential difference (Table 3). Thus, methanolic extracts showed the strongest antioxidant activity in trapping ABTS and DPPH radicals, as well as the highest FRAP reduction ability (759.78 ± 19.08 µmol TE/g, 451.08 ± 24.45 µmol TE/g, and 332.28 ± 10.93 µmol TE/g, respectively). *F. ulmaria* extracts obtained with 70% (*v*/*v*) aq. ethanolic solution also demonstrated essential antioxidant activity. The antioxidant activity of ethyl acetate extracts was the lowest among the three isolates.

As can be seen from Table 3, methanolic extracts of *A. glutinosa* showed the most pronounced antioxidant activity in trapping ABTS and DPPH radicals, as well as the most essential capacity for FRAP reduction (1094.02 ± 14.53 μmol TE/g, 584.45 ± 35.3 μmol TE/g, and 471.63 ± 7.06 μmol TE/g, respectively). All types of antioxidant activity observed with ethyl acetate extracts of *A. glutinosa* were minimal, if not negligible, for all isolates.

## 3. Discussion

It was found that the leaves of meadowsweet plants growing in other regions of Russia (e.g., in Samara region) contain aromatic compounds; in particular, phenylpropanoids—alcohols, aldehydes, acids and their derivatives [33,34,35]. Its pollen contains essential amino acids, triterpene acids, carotenoids (β-carotene), ascorbic acid, phenolcarboxylic acids (chlorogenic acids), and flavonoids, including catechins. The fruits of this plant contain tannins and flavonoids, whereas the seeds contain tannins, fatty oils and wax. Rhizomes and roots of *F. ulmaria* contain tannins, phenolic compounds, phenolic glycosides, flavonoids, phenolic acids, coumarins and vitamin C [18,36,37,38,39].

The seedlings of *A. glutinosa* growing in the forest-steppe zone of Central Russia are known for high contents of sterolds along with tannins and gallic acid [40]. The leaves of *A. glutinosa* contain flavonoid glycosides, phenolic acids (benzoates and phenylpropanoids), pyrocatechin acids, and triterpenoids [40].

Previous studies reported the antioxidant activity of the isolates, prepared from the aerial parts of *F. ulmaria*, which can be explained by the relatively high contents of flavonoids, tannins, phenolic glycosides (salicylate derivatives), volatile oils, minerals and vitamin C in this plant [41,42]. Remarkably, these high polyphenol contents were confirmed for both the roots and leaves of meadowsweet [27,43].

This study and the previous ones revealed similar patterns of secondary metabolites and their contents present in *A. glutinosa* and *F. ulmaria* growing in different regions of Russia. The most pronounced amounts of polyphenols and flavonoids were observed in methanol extracts of *A. glutinosa* and *F. ulmaria* growing in the Kaliningrad region, hence, methanol extracts of these plants exhibited the greatest antioxidant activity.

In the study of Dahija and co-workers [44], the total contents of phenolic acids and flavonoids along with the antioxidant activities of methanolic extracts of leaves and bark of three Alnus species were determined. The total phenolic contents in the extracts were determined spectrophotometrically by the Folin–Ciocalto method. The antioxidant activities of the extracts were determined by the consumption of the 1,1-diphenyl-2-picrylhydrazyl radical. The extract of *A. glutinosa* bark contained the highest amounts of total phenols (780 mg CAT/g), while the *A. glutinosa* leaf extract had the highest amount of flavonoids (30.01 mg RUT/g). All extracts showed higher antioxidant activity than thymol, which was used as a positive control.

Our findings are in agreement with the findings of previous studies [45]. The study of the chemical composition of the above-ground part of the meadowsweet (*F. ulmaria*) [45] showed the presence of simple phenols, flavonoids, organic acids, coumarins, tannins, anthracene derivatives, saponins, polysaccharides, carotenoids, amino acids, as well as macro- and microelements.

It was established [46] that aqueous and alcoholic extracts of the aerial parts of the meadowsweet exhibit pronounced antioxidant activity in the model reaction of oxygen electroreduction: all samples reacted with reactive oxygen species. As can be seen from the presented data, the antioxidant activity of aqueous ethanol extracts increases with increasing ethanol concentration. Thus, 70% and 95% ethanol extracts exhibited the strongest antioxidant action. In light of these findings, the authors investigated the chemical composition of a 70% water-ethanol extract of above-ground part of *F. ulmaria* in further depth. This analysis revealed the presence of the following groups of biologically active substances and individual compounds, which were identified by chromatographic mobility data in comparison with the corresponding reference samples: simple phenols (rhododendrol), flavonoids (quercetin, dihydroquercetin, apigenin, isoquercitrin, hyperoside, rutin), organic acids (m-hydroxybenzoic, salicylic, anisic, benzoic, gallic, gentisic, ferulic, chlorogenic, caffeic and quinic acids), coumarins (esculetin), hydrolysable tannins, steroidal saponins, and amino acids.

The antioxidant activity of plants is attributed to their composition and mixture of various antioxidants, primarily polyphenolic compounds with various mechanisms of action [47]. Due to their synergistic interaction, several methods must be used to determine the antioxidant capacity of plant extracts. DPPH and ABTS+ absorbance capacity are the most commonly used methods for determining antioxidant activity. Studies on the activity of DPPH and ABTS+ to remove radicals were performed to evaluate the antioxidant capacity of various active fractions compared to ascorbic acid (vitamin C), which was used as a control. Compared with other active components, the compound 2α,3β-dihydroxy-urs-12-en-28-aldehyde has better antioxidant activity, which is associated with the structure of ursane-type compounds [47]. Another explanation is that the molecule with the aldehyde group was more prone to oxidation processes and hence had higher antioxidant capability. Previous studies have confirmed the antioxidant potential of various meadowsweet extracts. When evaluating the activity of plants from related subgenera, it was discovered that crude extracts from various sources have strong antioxidant activity [47].

The antioxidant activity of *A. glutinosa* and *F. ulmaria* extracts was high, with *A. glutinosa* extracts being more active than *F. ulmaria* extracts. The methanol extract of *A. glutinosa* had the highest ABTS antioxidant activity (1094.02 ± 14.53 µmol TE/g). An aqueous extract of *A. glutinosa* also demonstrated a high ABTS radical scavenging activity (579.07 ± 41.87 µmol TE/g). Furthermore, methanolic extracts had the highest DPPH radical scavenging activity and FRAP reducing ability among *A. glutinosa* (584.45 ± 35.31 µmol TE/g and 471.63 ± 7.06 µmol TE/g, respectively), which is consistent with total extract yields and ellagic acid content in extracts. Aqueous extracts of *A. glutinosa* showed significant activity as well. Ethyl acetate extracts had a low antioxidant activity. Thus, methanol and 70% ethanol samples [46,47] of *A. glutinosa* and *F. ulmaria* extracts exhibit the highest antioxidant activity, which was confirmed by the results of our studies.

According to the study of Lauberts and co-workers [48], phenolic compounds extracted from *A. glutinosa* include bioactive components with a wide range of beneficial properties for human health, such as antioxidant, antibacterial, and anti-inflammatory properties. Despite its current use as a low-value fuel source, this article [48] discussed the potential value of *A. glutinosa* bark and cones for the production of biologically active compounds. Most of the currently available extraction methods use pure organic solvents to produce extracts with high antioxidant potential from bark and cone biomass. Taking advantage of accelerated solvent extraction, it has been demonstrated that the use of deionized water has the potential to replace organic solvents. In the case of a single-stage aqueous extraction, the total content of polyphenols in the extracts ranges from 0.55 to 0.62 GAE g/g depending on temperature, while as a result of sequential extraction with organic solvents, the content of polyphenols in 40% ethanol extracts ranges from 0.39 to 0.61 GAE g/g depending on the temperature. The influence of the total content of polyphenols and the total content of proanthocyanidins on antioxidant activity was demonstrated. Antioxidant activity (IC_50_, mg/L) of extracts obtained with organic solvents, in terms of (2,2-diphenyl-1-picrylhydrazyl) DPPH• varies from 4.05 to 9.58 depending on temperature within the range of 70–150 °C, respectively, while the results obtained with deionized water showed promising results in the range of 6.33–7.36 in the temperature range of 70–150 °C, respectively. Extraction with deionized water revealed that approximately 90% of the substances in the extracts obtained with organic solvents by sequential extraction can be obtained as deionized water extracts.

In a study of Altınyay et al. [49], aqueous and methanolic extracts of *A. glutinosa* were evaluated for their wound healing, anti-inflammatory, and antioxidant activities. In vivo wound models with linear incision and circular excision were created. Antioxidant activity was assessed using the effect of scavenging DPPH and ABTS radicals, power reduction, and denaturation of 2-deoxyribose targeting the nonspecific hydroxyl radical. The methanolic extract of *A. glutinosa* cones, the most potent extract, was fractionated using a bioassay guided fractionation method. NMR and IR analyses revealed that the isolated compound structure was shikimic acid.

The use of *A. glutinosa* extracts enhanced wound tension by 42.79% and provided 51.58% contraction. Wound tension, contractility, and tissue hydroxyproline levels were increased with use of EtOAc:MeOH (Fr. D) fraction, subfraction D27-38, and shikimic acid. In the acetic acid-induced capillary permeability inhibition assay, MB, Fr. D, D27-38 subfraction, and shikimic acid inhibited permeability with significant inhibition values of 30.22%, 32.46%, 38.24%, and 27.19%, respectively. In a model of carrageenan-induced hindpaw edema, MB showed 29.1% inhibition. Similarly, subfraction D27-38 and shikimic acid demonstrated remarkable anti-inflammatory and antioxidant effects. Shikimic acid showed a significant inhibitory effect (38.24%) on the hyaluronidase enzyme. According to the research results, shikimic acid is the primary compound responsible for antioxidant activity [49].

An analysis of the literature data [48,49] led us to the conclusion that phenolic compounds isolated from *A. glutinosa* by the method of methanol or ethanol extraction possess pronounced antioxidant properties, which was also confirmed in our studies. On the other hand, the aqueous extracts of *A. glutinosa* did not show any pronounced antioxidant, antibacterial, or anti-inflammatory properties [48].

## 4. Materials and Methods

### 4.1. Reagents

The individual phenolic components were identified by characteristic retention times and spectra of corresponding authentic standards. The following analytical standards were used: caftaric acid (CAS 67879-58-7, analytical standard), chlorogenic acid (CAS 327-97-9, ≥95.0%), trans-caffeic acid (CAS 501-16-6, analytical standard), *p*-coumaric acid (CAS 501-98-4, ≥98.0%), trans-ferulic acid (CAS 537-98-4, analytical standard), chicoric acid (CAS 6537-80-0, ≥ 95.0% HPLC grade), rosmarinic acid (CAS 20283-92-5, 96.0%), apigenin-7-*O*-glucoside (CAS 578-74-5, 93.47%), acacetin (CAS 480-44-4, ≥95% HPLC grade), 3,4-dihydroxybenzoic acid (protocatechuic acid, CAS 99-50-3, ≥97.0%), quercetin-3-*D*-glucoside (CAS 482-35-9, ≥90.0% (HPLC)), luteolin-7-glucoside (cynaroside, CAS 5373-11-5, analytical standard), hyperoside (CAS 482-36-0, analytical standard), rutin hydrate (CAS 207671-50-9, ≥98.0%), astragalin (kaempferol-3-glucosid, CAS 480-10-4, 92.5%), ellagic acid (CAS 476-86-4, ≥95.0% (HPLC)), (+)-catechin (CAS 154-23-4, ≥99.0% HPLC grade), and gallic acid (CAS 149-91-7, analytical standard).

AG Analytekspert, Moscow, Russia, supplied all standards and reagents with purity not less than chemically pure.

### 4.2. Objects of Research

Medicinal plants of the Kaliningrad region (*Filipendula ulmaria, Alnus glutinosa*) and their extracts were chosen as objects of study. The leaves of *F. ulmaria* and the female catkins of *A. glutinosa* were collected in the period June–October 2021 in the Kaliningrad region. The taxonomic identity of the plant material was confirmed according to the protocol # 8/2021 by Dr. Pungin, the head of the herbarium at the Institute of Living Systems of the I. Kant Baltic Federal University.

### 4.3. Extraction

To address the patterns of the plant secondary metabolites, three types of extracts were obtained from the cones of *F. ulmaria* and *A. glutinosa*: methanol by the Soxhlet method for 6 h (11 cycles), ethyl acetate by the Soxhlet method for 6 h (11 cycles) and purified water by maceration in a boiling water bath under reflux for 30 min, followed by a 10 min infusion (for *A. glutinosa*) and a 70% ethanol maceration in a boiling water bath under reflux for 60–90 min.

### 4.4. Determination of the Total Yield of Extracts

The total yields of the extracts were determined gravimetrically. The obtained extracts were concentrated in a vacuum rotary evaporator and dried in a Labconco Triad freeze dryer (Labconco, Kansas City, MO, USA).

### 4.5. Qualitative and Quantitative Analysis of Phenolic Compounds in Plant Extracts by HPLC

The RP-HPLC analyses were accomplished with LC-20AB Shimadzu Prominence chromatograph (Shimadzu, Kyoto, Japan) equipped with a binary pump, diode array detector SPD-M20A (Shimadzu, Kyoto, Japan) and a RP column Zorbax 300SB-C18 4.6 × 250 mm 5 µm (Agilent, Santa Clara, CA, USA). The separation was carried out at 40 °C in the gradient elution mode. Mobile phase: eluent A—0.1% (*v*/*v*) TFA in bi-distilled water, B—acetonitrile with trifluoroacetic acid. The flow rate was set to 1 mL/minute, and the UV data were acquired at 254, 280, and 325 nm.

The concentrations of individual compounds in the extracts were determined by external standardization. The determination accuracy was 3–7%.

### 4.6. Determination of Antioxidant Activity

Antioxidant activity (AOA) of extracts of plant samples was determined by the ability to scavenge free radicals of DPPH (2,2-diphenyl-1-picrylhydrazyl) and ABTS (2,2/-azino-bis(3-ethylbenzothiazoline-6-sulfonic acid), as well as by reducing the power in interaction with the Fe(III)-2.4.6-tripyridyl-s-triazine (FRAP) complex according to [21] with some modifications.

When determining antioxidant activity by the DPPH method, 20 µL of a plant extract or a standard solution was mixed with 300 µL of a freshly prepared 0.1 mmol/L solution of 2,2-diphenyl-1-picrylhydrazyl. The mixture was incubated in the dark at room temperature for 30 min. The decrease in absorbance compared to the control (solvent used for extraction) was recorded at 515 nm.

The solution of the ABTS radical was prepared directly before analysis. The ABTS radical was generated by mixing aliquots of 7.0 mmol/L ABTS solution and 2.45 mmol/L potassium persulfate solution. The solution was kept for 16 h in the dark at room temperature (RT). To start the reaction, 20 µL of a plant extract or standard was added to 300 μL of the prepared solution of the ABTS+ radical cation. The absorbance was measured at 734 nm with a UV-1280 spectrophotometer (Shimadzu, Kyoto, Japan) after the mixture was incubated for 15 min at 37 °C in the dark. ABTS reagent and the appropriate solvent used for extraction were used as blanks.

To determine the antioxidant activity of the extracts, freshly prepared FRAP reagent was used, prepared by mixing 10 parts of 0.3 mol/L acetate buffer (pH 3.6), one part of a 10 mmol/L solution of 2,4,6-tripyridyl-s-triazine in 40 mmol/L HCl, and one part of an aqueous 20 mmol/L solution of FeCl_3_ × 6H_2_O. The reaction was started by mixing 300 µL of the FRAP reagent and 20 µL of the extract or standard solution. The reaction time was 10 min at 37 °C in the dark. The absorbance was measured at 593 nm with a UV-1280 spectrophotometer (Shimadzu, Kyoto, Japan). The FRAP reagent and the appropriate extraction solvent were used as blanks.

When measuring antioxidant activity using DPPH, ABTS, and FRAP methods, solutions of Trolox (6-hydroxy-2,5,7,8-tetramethylchroman-2-carboxylic acid) of known concentration 0.1 M were used as standard solutions. The results of the analyzes were expressed in µmol Trolox equivalents per gram of plant dry weight (µmol TE/g).

All spectrophotometric measurements were performed using a CLARIOstar microplate reader (BMG Labtech, Ortenberg (Hesse), Germany).

### 4.7. Statistical Analysis

The data were subjected to analysis of variance (ANOVA) using Statistica 10.0 (StatSoft Inc., 2007, USA). Significance of the observed differences was assessed by Post hoc analysis (Duncan’s test). The equality of the variances of the extracted samples was checked using the Levene test. Differences between means were considered significant when the confidence interval is smaller than 5% (*p* < 0.05).

## 5. Conclusions

Plant-derived antioxidants are believed to be the most promising for continuous therapeutic and dietary consumption, as they are typically less toxic to humans than synthetic antioxidants like dibunol, probucol, cystalite, and mexamine, which are not promising, therefore, in clinical practice [50].

Aqueous ethanolic extracts of the *F. ulmaria* showed the most abundant patterns of phenolic metabolites in this study. Methanolic extracts delivered promising results as well. The methanolic extract of *A. glutinosa* contained the highest concentration of ellagic acid. Approximately half the amount of this acid was found in the water extract of *A. glutinosa*. On the other hand, ethyl acetate extracts for both plants showed the lowest yield of phenolic components.

The antioxidant activity of the extracts of *A. glutinosa* and *F. ulmaria* was found to be strong, with *A. glutinosa* extracts being more active than *F. ulmaria* extracts. As a result of the activation of free radical processes, oxidative modification of various biomolecules (lipids, proteins, nucleic acids) occurs. This ultimately leads to damage and death of cells in tissues and organs [50]. As weakened antioxidant defense mechanisms play a key role in enhancing free-radical reactions, one of the most pressing biomedical research priorities is the development of drugs and biologically active food supplements with antioxidant properties derived from *F. ulmaria* and *A. glutinosa*, with the goal of using them to slow down human body aging. This creates good prospects for the further use of *F. ulmaria* and *A. glutinosa* extracts as a source of natural antioxidants.

## Figures and Tables

**Table 1:** Total yields of plant extracts.

Plant	Extract Yield, wt. %			
	EtAc	MeOH	70% (*v*/*v*) aq. EtOH	H_2_O
*A. glutinosa* (cones)	4.59 ± 0.18 ^a^	31.32 ± 1.9 ^b^	-	25.71 ± 1.8 ^c^
*F. ulmaria* (leaves)	6.23 ± 0.18 ^a^	23.03 ± 1.8 ^b^	26.87 ± 1.8 ^b^	-

Values in row followed by the same letter do not differ significantly (*p* < 0.05) as assessed by the post hoc test (Duncan’s test). Data presented as a mean ± SD (*n* = 3).

**Table 2:** The contents of individual phenolic compounds in *F. ulmaria* and *A. glutinosa* extracts.

Biologically Active Substances	Release Time, min	Contents (g/kg)					
		*F. ulmaria* (Leaves)			*A. glutinosa* (Cones)		
		EtAc	MeOH	70% (*v*/*v*) aq. EtOH	EtAc	MeOH	H_2_O
Hyperoside	18.7–19.4	0.685 ± 0.09 ^a^	3.781 ± 0.30 ^b^	4.920 ± 0.30 ^b^	-	-	-
Ellagic acid	18.2	-	0.730 ± 0.09 ^a^	1.250 ± 0.20 ^a^	10.994 ± 1.09 ^b^	44.883 ± 5.18 ^c^	21.832 ± 2.09 ^d^
Quercetin-3D-glucoside	19.6–20.3	0.061 ± 0.09 ^a^	1.134 ± 0.20 ^b^	1.659 ± 0.20 ^b^	-	-	-
Luteolin-7-glucoside	21.2–21.8	0.242 ± 0.07 ^a^	1.798 ± 0.20 ^b^	2.124 ± 0.20 ^b^	-	-	-
Astragalin	25.1	0.222 ± 0.07 ^a^	0.767 ± 0.09 ^a^	1.071 ± 0.19 ^a^	-	-	-
Coumaric acid	14.6	-	-	0.038 ± 0.001	-	-	-
Caftaric acid	8.4	-	0.064 ± 0.09 ^a^	0.112 ± 0.05 ^a^	-	-	-
Chlorogenic acid	10.2–10.4	trace	0.342 ± 0.08 ^a^	0.423 ± 0.06 ^a^	-	-	-
Gallic acid	3.8–3.9	0.263 ± 0.07 ^a^	0.870 ± 0.09 ^a^	0.212 ± 0.05 ^a^	0.141 ± 0.05 ^a^	0.919 ± 0.09 ^a^	2.972 ± 0.09 ^b^
Catechin	9.6	-	0.598 ± 0.08	-	-	-	-
3,4-Dihydroxybenzoic acid	5.2–5.6	0.014 ± 0.05	-	-	trace	trace	-
Rutin	19.2	0.377 ± 0.08	-	-	-	-	-

The analysis relied on RP-HPLC-UV with detection at 254 nm Values in row followed by the same letter a, b, c or d do not differ significantly (*p* < 0.05) as assessed by the post hoc test (Duncan’s test). Data presented as a mean ± SD (*n* = 3).

**Table 3:** Antioxidant activity of the extracts obtained from the aerial parts of in *F. ulmaria* and *A. glutinosa* extracts.

Plant	Extractant	Antioxidant Activity		
		ABTS	DPPH	FRAP
*F. ulmaria* (leaves)	70% (*v*/*v*) aq. EtOH	243.00 ± 14.70 ^a^	284.25 ± 20.17 ^a^	190.75 ± 11.48 ^a^
	EtAc	91.01 ± 3.22 ^b^	32.88 ± 0.26 ^b^	22.93 ± 1.05 ^b^
	MeOH	759.78 ± 19.08 ^c^	451.08 ± 24.45 ^c^	332.28 ± 10.93 ^c^
*A. glutinosa* (cones)	H_2_O	579.07 ± 41.8 ^a^	275.89 ± 23.55 ^a^	378.69 ± 31.03 ^a^
	EtAc	103.90 ± 0.61 ^b^	48.12 ± 2.95 ^b^	36.81 ± 1.06 ^b^
	MeOH	1094.02 ± 14.53 ^c^	584.45 ± 35.3 ^c^	471.63 ± 7.06 ^c^

Values in columns followed by the same letter a, b or c do not differ significantly (*p* < 0.05) as assessed by the post hoc test (Duncan’s test). Data presented as a mean ± SD (*n* = 3).

## Data Availability

The data are included in the manuscript.
